# The protective effect of *Bifidobacterium bifidum* G9-1 against mucus degradation by *Akkermansia muciniphila* following small intestine injury caused by a proton pump inhibitor and aspirin

**DOI:** 10.1080/19490976.2020.1758290

**Published:** 2020-06-09

**Authors:** Tsutomu Yoshihara, Yosuke Oikawa, Takayuki Kato, Takaomi Kessoku, Takashi Kobayashi, Shingo Kato, Noboru Misawa, Keiichi Ashikari, Akiko Fuyuki, Hidenori Ohkubo, Takuma Higurashi, Yoko Tateishi, Yoshiki Tanaka, Shunji Nakajima, Hiroshi Ohno, Koichiro Wada, Atsushi Nakajima

**Affiliations:** a Department of Gastroenterology and Hepatology, Yokohama City University School of Medicine, Yokohama, Japan; b R&D Center, Biofermin Pharmaceutical Co., Ltd., Kobe, Japan; c Department of Gastroenterology, International University of Health and Welfare Atami Hospital, Atami, Japan; d Department of Pathology, Yokohama City University Hospital, Yokohama, Japan; e Department of Pharmacology, Faculty of Medicine, Shimane University, Izumo, Japan

**Keywords:** Proton pump inhibitor, aspirin, intestinal injury, *Bifidobacterium bifidum* G9-1, *Akkermansia muciniphila*

## Abstract

Background

Proton pump inhibitors (PPIs) can alleviate upper gastrointestinal injury but paradoxically exacerbate aspirin (ASA)-induced small intestine injury. In this study, our goal was to simulate this exacerbation by developing an appropriate animal model, which may help in establishing treatments. **Methods**: Male mice were fed a 60% fructose diet for 9 weeks, then administered 200 mg/kg ASA 3 h before sacrifice. The PPI omeprazole was administered intraperitoneally once daily for 9 weeks. *Bifidobacterium bifidum* G9-1 was administered orally for the last week. In addition, *Akkermansia muciniphila* was administered orally for 9 weeks instead of omeprazole. **Results**: ASA-induced small-intestine injury was observed in high-fructose fed mice. Omeprazole exacerbated ASA-induced intestinal damage, significantly decreased *Bifidobacteria* levels, and significantly increased *A. muciniphila* counts in the jejunum. The direct administration of *A. muciniphila* caused thinning of the jejunum mucus layer, which was also observed in mice that received ASA and omeprazole. On the other hand, the administration of *Bifidobacterium bifidum* G9-1 inhibited *A. muciniphila* growth and reduced thinning of the mucus layer. The number of goblet cells in the jejunum was reduced by the administration of ASA and omeprazole, while *Bifidobacterium bifidum* G9-1 prevented the reduction. **Conclusions**: These results suggest that omeprazole-induced gut dysbiosis promotes *Akkermansia* growth and inhibits *Bifidobacterium* growth, leading to a thinning of the mucus layer through a reduction in goblet cells in the small intestine. Probiotics are, therefore, a promising approach for the treatment of small intestine injury.

## Introduction

Although aspirin (ASA) is widely used to prevent cardiovascular diseases, it can injure both the small intestine and the stomach. In the small intestine, ASA causes mucosal damage that in some cases can progress to intestinal bleeding.^1–3^ While its adverse effects may result in discontinuation of ASA, no effective preventative or treatment measures have yet been established.

The incidence of cardiovascular disease and the morbidity of diseases associated with a western-style diet have risen over the past few decades. Although the trend differs by geographical region, the number of deaths from cardiovascular disease is still increasing globally.^4^ We hypothesized that some aspects of the western diet may be associated with ASA-induced small intestine injury, particularly the overconsumption of fructose sugars contained in beverages and snacks. Since a high-fructose diet can lead to obesity and metabolic syndrome, it has become a societal problem. The overconsumption of a high-fructose diet can also contribute to nonalcoholic fatty liver disease (NAFLD) and diabetes, as well as increase intestinal permeability and serum endotoxin levels.^5–7^

Proton pump inhibitors (PPIs) have been used to prevent ASA-induced upper gastrointestinal mucosal injury and are considered to be very safe. However, because PPIs induce gut dysbiosis, their use is a potential risk factor for ASA-induced small intestine injury,^2,8^ although the mechanism that accounts for this risk is unclear.

Creating mouse models of ASA-induced small intestine injury has been difficult, as the administration of ASA does not induce mucosal injury in mice.^1,9^ By focusing on the effect of a high-fructose diet on intestinal permeability, we were able to establish a mouse model capable of assessing the effects of PPIs.

The pathophysiology and treatment of PPI-induced gut dysbiosis have been closely examined. Wallace et al. have reported that PPIs reduce *Bifidobacterium* levels and exacerbate Naproxen-induced small intestine injury.^9^ However, the relationship between *Bifidobacterium* and small intestine injury is not clearly understood.

Probiotics have long been used to improve bowel function. While some clinical trials have reported probiotics to be effective in treating ASA-induced small intestine injury, the mechanisms of action have not been clarified.^10,11^ The G9-1 strain of *Bifidobacterium bifidum* (hereafter referred to as G9-1) was isolated from a healthy individual and has been safely used to ameliorate multiple bowel symptoms.^12^ By bolstering the mucosal barrier, it alleviates drug-induced small intestine injuries such as those caused by NSAIDs.^13^ These findings suggest the potential of G9-1 to improve ASA-induced small intestine injury and PPI-induced gut dysbiosis.

In this study, we investigate another key player, *Akkermansia muciniphila*, whose role is degrading mucin in intestines. Goblet cells secret mucin onto the intestinal surface, where there is a large amount of Trefoil factor family-3 (TFF3) protein. TFF3 induces cell migration into wounds, and it is essential for the rapid repair of mucosal injury.^14^ In mice lacking TFF3, dextran sulfate sodium (DSS) causes severe colitis.^15^

Here, we assessed the adverse effects of the PPI omeprazole, as well as the effects of G9-1, in a mouse model. Clarifying the mechanisms responsible for the development of intestinal injury and identifying therapeutic methods are extremely important for patients suffering from intestinal bleeding due to the combined use of ASA and PPIs. Importantly, patients suffering from both cardiovascular diseases and intestinal bleeding would be able to continue using ASA as a treatment for their cardiovascular diseases.

## Results

### Establishment of a mouse model of ASA-induced small intestine injury

**Figure 1.**
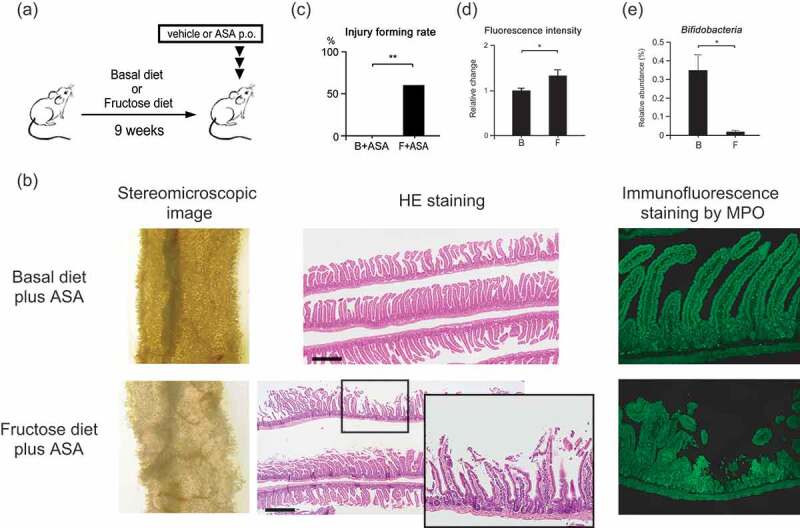
Mouse model of ASA-induced small intestine injury. Male C57BL/6JJcl mice (2–4) were housed in each cage. (a) Protocol for the model. Basal or 60% fructose diets were fed to six-week-old mice for 9 weeks. On the final day, ASA or vehicle was administered orally 3 h before sacrifice. (b) Macroscopic and microscopic images of the upper jejunum assayed by stereomicroscopic imaging, HE staining, and by immunofluorescence staining for MPO. Scale bars = 500 μm. (c) Rates of small intestine injury formation (n = 10–11 per group). (d) Small intestinal permeability. Both groups were administered vehicle instead of ASA (n = 8 for each group). (e) Relative abundance of *Bifidobacteria* in fecal samples. Both groups were administered vehicle instead of ASA (n = 5 for each group). Data are means ± SEM. *p < .05, Fisher’s exact tests (c), Welch’s *t*-test (d), or Wilcoxon rank-sum test (e). B, Basal diet; F, High-fructose diet.

Mice were fed a high-fructose diet or a basal diet for 9 weeks and then treated with vehicle or ASA (Figure 1a). Compared to the basal diet group, a deficiency in the upper villi was observed in the jejunum of mice in the high-fructose diet group (Figure 1b, c). Similarly, the high-fructose diet with the vehicle group had increased intestinal permeability compared with the basal diet with the vehicle group (Figure 1d). Immunofluorescence staining confirmed the presence of MPO-positive cells around damaged areas. The levels of *Bifidobacteria* were significantly lower in the high-fructose diet group compared to the basal diet group (Figure 1e). In this analysis, *Bifidobacterium pseudolungum* subsp. *globosum* was mainly detected. However, we used *Bifidobacterium bifidum* G9-1 in this study because it is widely used as a probiotics in humans, and it could be used in clinical pathology in ASA-induced small intestine injury. These results showed that the high-fructose diet changes the microbiota profile, loosens the intestinal barrier, decreases intestinal *Bifidobacteria* levels, and promotes ASA-induced small intestine injury.

**Figure 2.**
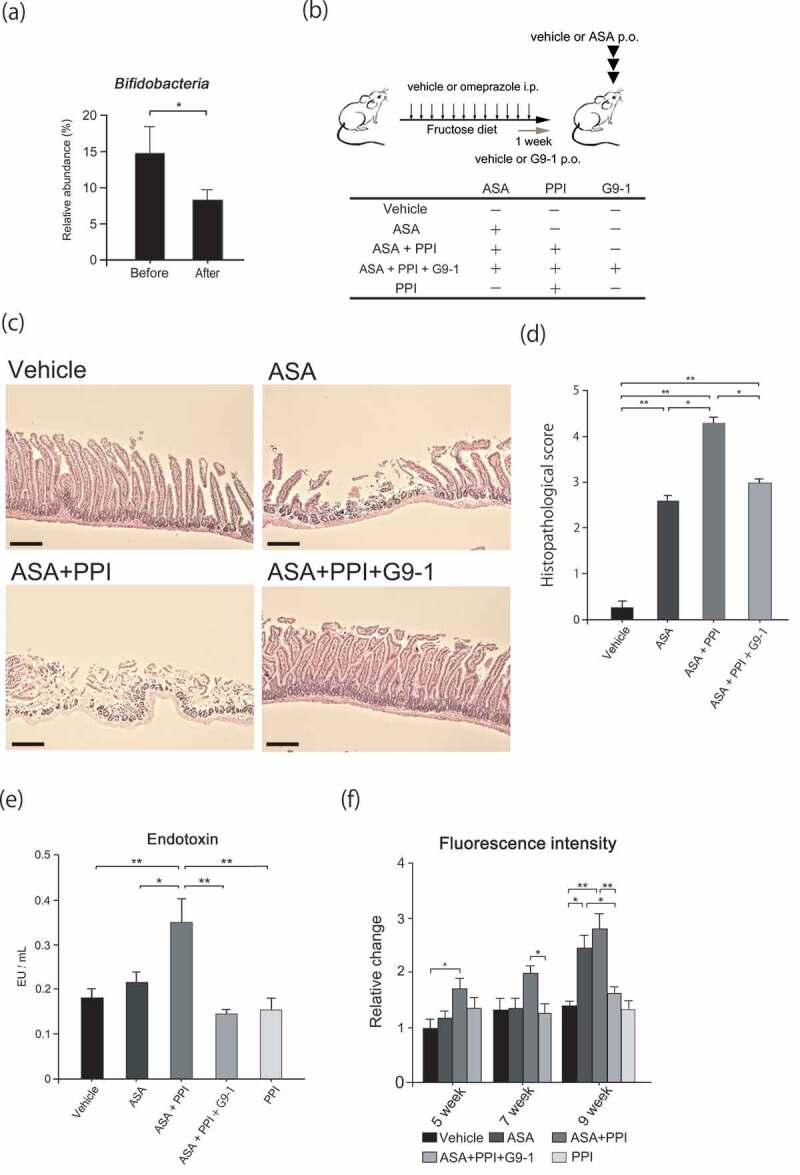
Effect of PPI and *Bifidobacterium bifidum G9-1* in the mouse model of ASA-induced injury. Male C57BL/6JJcl mice (2–4) were housed in each cage. (a) Relative abundance of *Bifidobacteria* in human fecal samples (n = 19) before and after omeprazole treatment. (b) Protocol for the mouse model. Sixty percent of fructose diets were fed to six-week-old mice for 9 weeks. Mice were divided into five treatment groups. Omeprazole or vehicle was administered intraperitoneally once daily. G9-1 or vehicle was administered orally for 1 week before sacrifice. On the last day, ASA or vehicle was administered orally 3 h before sacrifice. (c) HE staining of the jejunum. Scale bars = 100 μm. (d) Histopathological scores for small intestine injury. (n = 8–11 per group). (e) Levels of serum endotoxin at the end of the experiment (n = 5–8 per group). (f) Small intestinal permeability at five, seven, and nine weeks (n = 5–8 per group). Data are means ± SEM. *p < .05, **p < .01, Wilcoxon signed-sum test (a), Steel-Dwass test (d), or Tukey–Kramer test (e, f).

When healthy human subjects were treated with omeprazole (hereafter referred to as PPI), the relative abundance of *Bifidobacteria* in fecal samples decreased by about 40%. A principle coordinates analysis (PCoA) showed significant changes in gut microbiota clustering (PERMANOVA *p* < .05, Figure 2a and Figure S1), strongly indicating that PPI causes gut dysbiosis.

Since a reduction in *Bifidobacteria* reportedly correlates with exacerbation of small intestine injury caused by PPIs,^9^ the effects of PPI and G9-1 were assessed in our mouse model. The PPI alone group was not included because PPIs did not cause the injury (data not shown). The jejuna of mice in the ASA and ASA+PPI groups were damaged, but this damage was attenuated in the ASA+PPI+G9-1 group. Furthermore, the ASA+PPI group had a significantly higher pathology score than the ASA group, whereas the ASA+PPI+G9-1 group had a score significantly lower than the ASA+PPI group. These results indicate that intestinal injury is exacerbated by PPI and alleviated by G9-1 (Figure 2c, d). Furthermore, the endotoxin levels in the ASA+PPI group were significantly increased over those of all other groups (Figure 2e).

A significant increase in intestinal permeability was observed in the ASA group compared to the vehicle group. While the administration of PPI alone did not increase intestinal permeability, the concomitant administration of ASA and PPI increased intestinal permeability, especially at week 5. These data suggest that PPI itself does not increase small intestinal permeability, but instead exacerbates the increases caused by ASA. Further administration of G9-1 significantly suppressed these increases at weeks 7 and 9 (Figure 2f).

### *PPI increases the abundance of* Akkermansia muciniphila *in the jejunum, causing thinning of the mucus layer*

To confirm the effect of PPI on gut dysbiosis, jejunum microbiota profiles were assessed using a Weighted Unifrac analysis. While the microbial profiles of the ASA+PPI and PPI groups were similar (PERMANOVA *p* = .15), they differed from the vehicle-only control group (PERMANOVA *p* < .01 vehicle control vs. PPI, *p* < .01 vehicle control vs. ASA+PPI). On the other hand, the profile of the ASA+PP1+ G9-1 group was similar to that of the vehicle group (PERMANOVA *p* = .08, Figure S2). Furthermore, significant changes in the relative abundance of *Akkermansia* and *Bifidobacteria* were observed (Figure 3a). These results demonstrate that G9-1 improves the intestinal dysbiosis caused by PPI.

**Figure 3.**
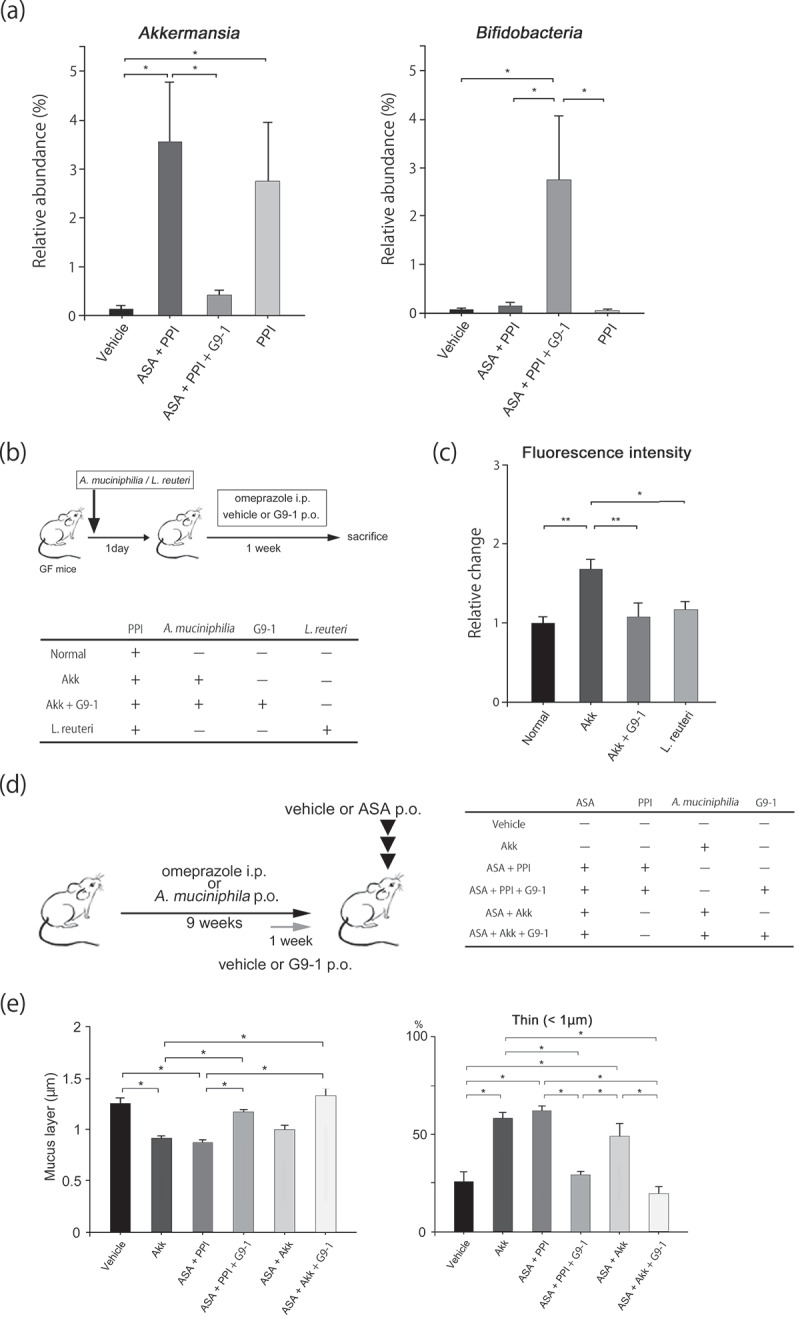
Effect of *Akkermansia muciniphila* and G9-1 on the mucus layer. Male C57BL/6JJcl mice (2–4) were housed in each cage. (a) Relative abundance of *Akkermansia* and *Bifidobacteria* in the jejunum of mice in the different treatment groups (n = 6–8 per group) at week 9. (b) Protocol used for the gnotobiotic trial. Germ-free male C57BL/6NJc1 mice were housed in a germ-free environment (n = 8 for each group). Six-week-old mice were fed a 60% fructose diet and divided into four treatment groups. On the first day, the mice received either *A. muciniphila* or *Lactobacillus reuteri* orally. From the second day, omeprazole was administered intraperitoneally every day. G9-1 was administered orally to the Akk+G9-1 group from the second day until the end of the study. (c) Small intestinal permeability in gnotobiotic mice (n = 8 for each group). (d) Protocol of the experiment used to investigate the effect of *A. muciniphila* on the small intestine injury. A 60% fructose diet was fed to six-week-old mice for 9 weeks, and divided into six treatment groups. Either omeprazole, *A. muciniphila*, or vehicle was administered once daily during the treatment period. G9-1 or vehicle was administered orally for 1 week before sacrifice. ASA or vehicle was administered orally 3 h before sacrifice. (e) Thicknesses of the mucus layer in the jejunum (left) and rates of mucus layers with thicknesses less than 1 μm (right). ImageJ was used to measure the thickness of the mucus layer, and samples were measured using approximately 124 points per sample (n = 5–6 per group). (f) PAS staining of the top of villi in the jejunum of mice in the different treatment groups. Scale bars = 10 μm. The arrows indicate the mucus layer. (g) The number of goblet cells in the jejunum of mice in the different treatment groups (n = 5–6 per group). Scale bars = 100 μm. Arrows indicate goblet cells. (h) Expression levels of *Tff3* in the jejunum (n = 8–12 per group). Data are means ± SEM. *p < .05, **p < .01, Steel-Dwass test (a, e, g), or Tukey–Kramer test (c, h). Akk, *Akkermansia muciniphila.*

**Figure 3.**
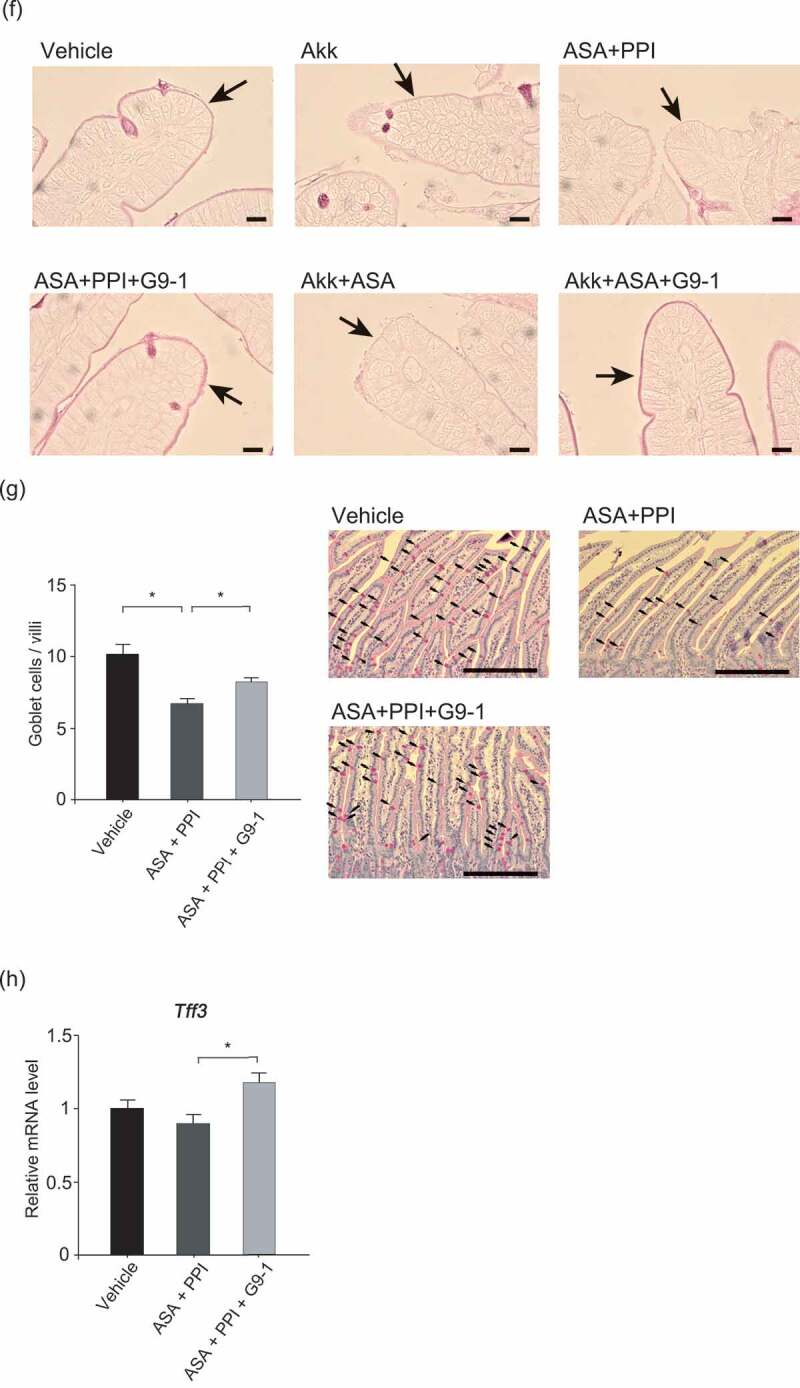
(Continued).

The relative abundance of *Akkermansia* in the jejunum in the ASA+PPI group was significantly higher compared to the vehicle control group. However, levels were significantly lower in the ASA+PPI+G9-1 group compared to the ASA+PPI group. Prior to being fed a high-fructose diet and drug administration, low levels of *Akkermansia* were observed in mice (Figure S3A). The administration of ASA alone did not change the relative abundance of *Akkermansia* (Figure S3B). Interestingly, *Akkermansia* did not increase when mice were fed the basal diet and administered omeprazole (Figure S3C), which indicates that a high-fructose diet is also an important factor for induction of *Akkermansia* overgrowth in the jejunum. To further investigate the effect of *Akkermansia*, its effect on intestinal permeability was examined using *Akkermansia muciniphila* (*A. mucinphila*) gnotobiotic mice. *Lactobacillus reuteri* was used as the control strain, since our data suggest that it did not display mucin-degrading capability (Figure S4), and we did not consider *L. reuteri* to increase intestinal permeability as it improves intestinal permeability due to colitis.^16^ Additionally, because the levels of *L. reuteri* were approximately equivalent to those of *A. muciniphila* in our murine models, we allowed *L. reuteri* to be used as the control. The small intestinal permeability of *A. muciniphila* gnotobiotic mice was significantly higher than that of the normal group (Figure 3c, *p* < .01). On the other hand, the intestinal permeabilities of the *Lactobacillus reuteri* gnotobiotic mice and *Akkermansia* (Akk) + G9-1 mice were similar to those in the normal group. These findings suggest that the presence of *A. muciniphila* increases small intestinal permeability.

*A. muciniphila* is known to degrade mucin.^17^ Thinning of the mucus layer was observed in the Akk, ASA+Akk, and ASA+PPI groups (*p* < .05 vs. vehicle control). In contrast, the thicknesses of the mucus layers in the ASA+PPI+G9-1 and ASA+Akk+G9-1 groups were similar to those in the vehicle group, indicating that the presence of G9-1 suppresses the thinning of the mucus layer caused by *A. muciniphila* (Figure 3e, f).

Since mucosal protection is promoted by mucin secretion from goblet cells and the expression of the TFF, we measured the numbers of PAS-positive goblet cells and the expression levels of *Tff3* in the jejunum. Goblet cells were counted only in undamaged areas of the jejunum to allow uniform comparisons between groups. Even with counting restricted to undamaged regions, goblet cells were less frequent in the ASA+PPI group than in the vehicle control, and were significantly increased by G9-1 (Figure 3g). The expression levels of *Tff3* in the ASA+PPI group were 11% lower than the vehicle group but were significantly increased by G9-1 (*p* < .05 vs. ASA+PPI). A trend of increased numbers of goblet cells and an increase in *Tff3* expression levels were also seen in mice fed the basal diet after administration of G9-1 (Figure S6); however, the changes did not reach the level of significance, possibly because the lack of ASA+PPI administration meant that the number of goblet cells remained high. These results indicate that G9-1 induces the expression of *Tff3*, thereby consolidating the mucus layer and alleviating small intestine injury concomitant with the administration of ASA+PPI.

### Small intestine injury promotes epithelial renewal and G9-1 regulates it

**Figure 4.**
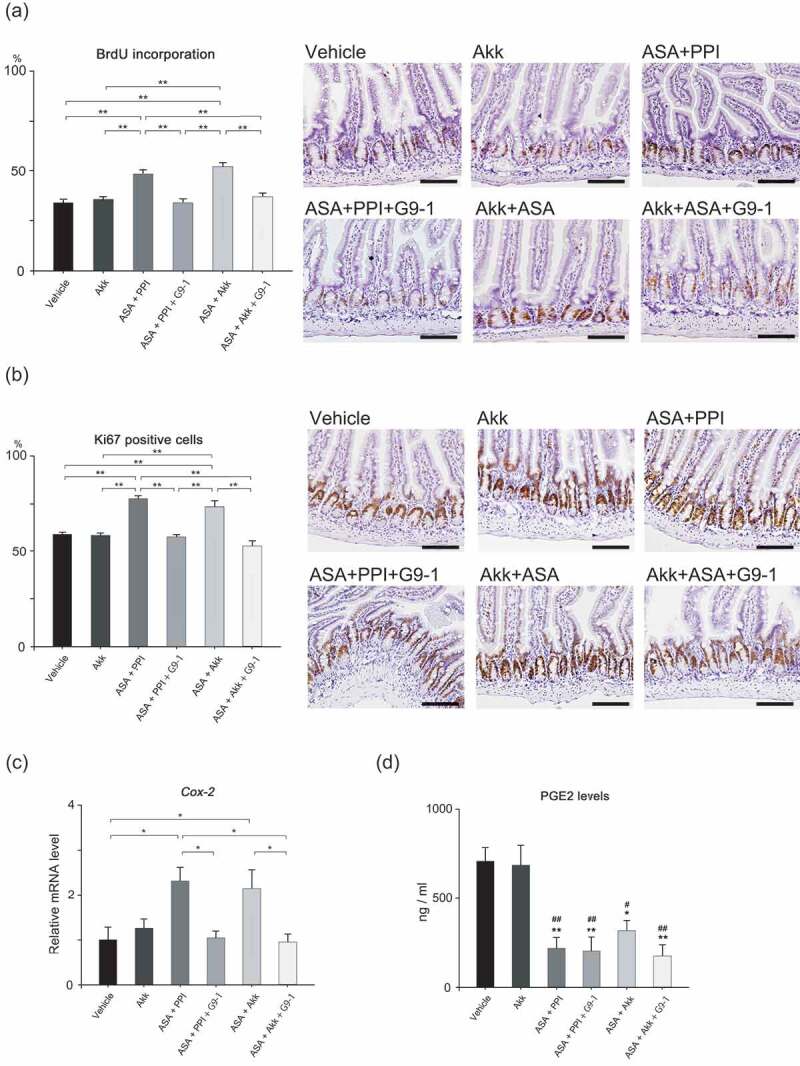
The epithelial renewal study, *Cox-2* gene expression, and COX-2 activity. (a) The incorporation of BrdU (n = 5 for each group). The total number of positive cells from 50 crypts was counted. Scale bars = 100 µm. (b) Ki67 staining (n = 5 for each group). The total number of positive cells from 50 crypts was counted. Scale bars = 100 µm. (c) Levels of *Cox-2* gene expression in the jejunum of mice in the different treatment groups (n = 6–7 per group). (d) Levels of PGE2 in the jejunum of mice in the different treatment groups by ELISA (n = 7 for each group). Data are means ± SEM. *p < .05, **p < .01, Tukey–Kramer test (a, b, c). *p < .05 vs. the Vehicle group, **p < .01 vs. the Vehicle group, #p < .05 vs. the Akk group, ##p < .01 vs. the Akk group, Tukey–Kramer test (d).

To investigate whether G9-1-dependent protection against small intestinal injury occurs due to increased epithelial renewal, we performed bromodeoxyuridine (BrdU) incorporation studies and performed staining for Ki67 as cell proliferation markers. Positive cells in the crypts of jejunum were counted and compared among the groups. All of the results showed the same trends (Figure 4a, b). Epithelial renewal was promoted in the ASA+PPI and Akk+ASA groups, and was not promoted by G9-1. These results indicate that epithelial repair was promoted when the intestine was severely injured, but was suppressed when the injury was ameliorated. Additionally, as cyclooxygenase-2 (COX-2) is known to promote cell proliferation, especially in a carcinogenesis model,^18^ we investigated the expression of the *Cox-2* gene. It was upregulated in the ASA+PPI and ASA+Akk groups, but not in the ASA+PPI+G9-1 and ASA+Akk+G9-1 groups (Figure 4c). This result is very similar to the cell proliferation study.

Furthermore, we performed a prostaglandin E2 (PGE2) enzyme-linked immunosorbent assay (ELISA) to investigate COX-2 activity. PGE2 was significantly lower in the ASA+PPI, ASA+PPI+G9-1, Akk+ASA, and Akk+ASA+G9-1 groups compared to that in the vehicle control and Akk groups. These results confirmed that ASA inhibited COX-2 activity and decreased PGE2 levels in the jejunum without inhibiting Cox-2 expression (Figure 4d).

### G9-1 decreases inflammatory chemokines/cytokines and induces regulatory T cells in the small intestine

**Figure 5.**
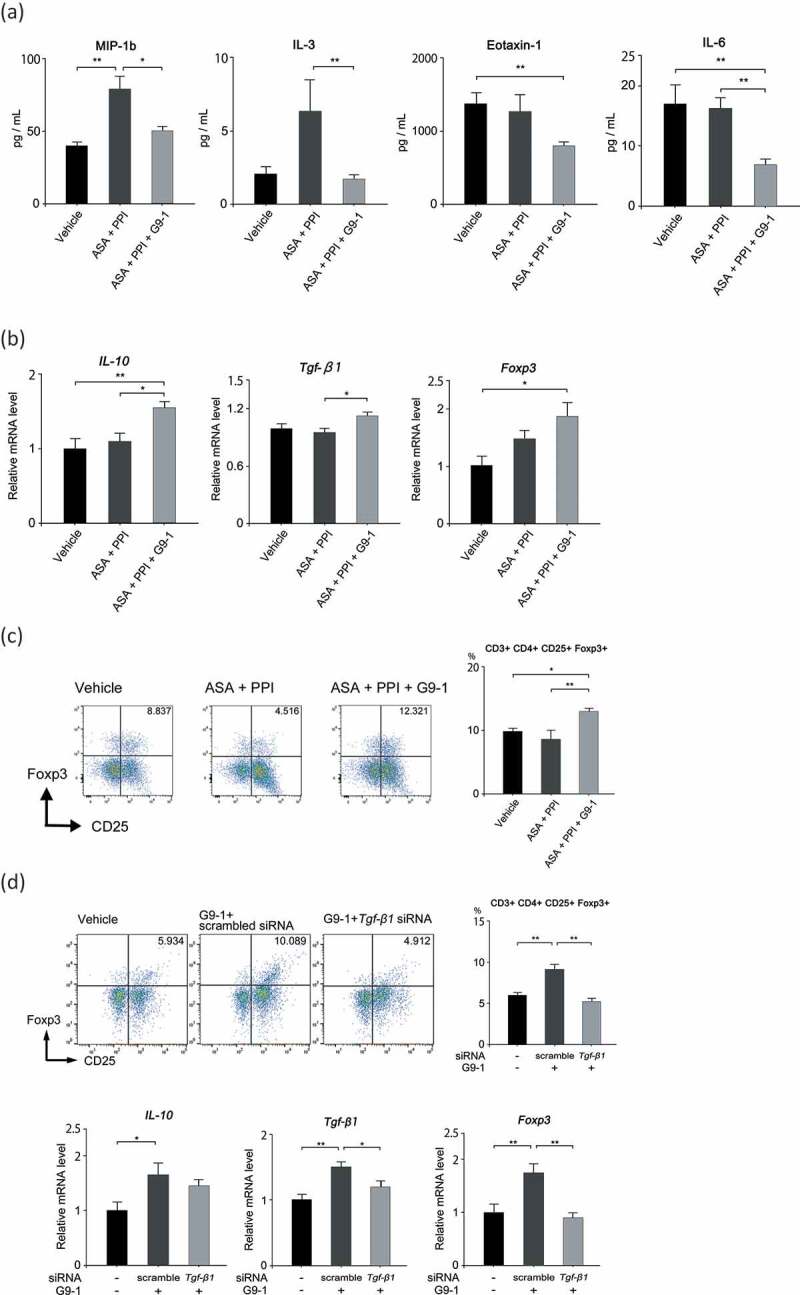
Effect of G9-1 on Treg induction-associated inflammatory cytokines and chemokines. (a) Expression levels of inflammatory cytokines and chemokines in the sera of mice in the different treatment groups (n = 5–10 per group). (b) Levels of *Il-10, Tgf-β*, and *Foxp3* gene expression in the jejunum of mice in the different treatment groups (n = 5–7 per group). (c) Flow cytometric analysis of isolated lamina propria cells. Flow was first gated on CD3^+^CD4^+^ cells and then on CD25^+^Foxp3^+^ cells (n = 4–5 per group). (d) Flow cytometric analysis of isolated lamina propria cells and levels of *Il-10, Tgf-β*, and *Foxp3* gene expression in the jejunum of mice. The *Tgf-β1* siRNA was transfected 48 h and 12 h before sacrifice. Scrambled siRNA was used as a control against the *Tgf-β1* siRNA (n = 8–10 per group for the flow cytometric analysis, n = 5–7 per group for the PCR analysis). Data are means ± SEM. *p < .05, **p < .01, Tukey-Kramer test (a–d).

To investigate the mechanism underlying the anti-inflammatory effect of G9-1, the serum levels of inflammatory cytokines and chemokines were assessed (Figure 5a). The levels of both MIP-1β and IL-3 were increased in the ASA+PPI group compared to vehicle control. Treatment with G9-1 decreased the levels of MIP-1β and IL-3 to levels similar to the vehicle control. Levels of IL-6 and eotaxin-1 were decreased, but only in the ASA+PPI+G9-1 group. Eotaxin-1 promotes the induction of eosinophils. In our model, neither ASA nor PPI were considered to be involved in allergic mechanisms. Therefore, we did not observe the increase of Eotaxin-1. On the other hand, the level of IL-6 may not immediately peak after the administration of ASA. It is reported that proteins such as NF-κB or IκBζ promote IL-6 expression, which generally occurs a few hours after these transcription factors are activated.^19,20^ We hypothesized that the time from the administration of ASA to sacrifice was too short to observe IL-6 upregulation.

Additionally, we analyzed gene expression in the jejunum and the distribution of regulatory T cells (Tregs) in small intestinal LPL. The expression levels of *Il10, Tgf-β1*, and *Foxp3* in the jejunum increased significantly with the administration of G9-1 (Figure 5b). Finally, flow cytometry showed that levels of CD25^+^/Foxp3^+^ T-cells were significantly increased in the ASA+PPI+G9-1 group (*p* < .01 vs. ASA+PPI) (Figure 5c). To confirm whether Treg induction was due to the effects of G9-1 effects, we performed additional experiments by using small interfering RNA (siRNA) against *Tgf-β1*. PCR analysis showed that the administration of siRNA downregulated *Tgf-β1* and *Foxp3* expression. Furthermore, it suppressed the increase in Treg levels (Figure 5d). These results suggest that one of the mechanisms underlying G9-1-alleviated small intestine injury may be the triggering of Treg differentiation.

## Discussion

Multiple groups have attempted to create an experimental animal model of NSAID-induced small intestine injury.^1^ However, there is a difference between NSAIDs and ASA, since models of NSAID-induced small intestine injury can easily be created. The major difference between ASA and other NSAIDs is that ASA has anti-platelet effects. ASA irreversibly inhibits COX-1, which consequently suppresses thromboxane A2. Due to the irreversible effect, the anti-platelet effect lasts for a few days.^21^ Furthermore, a capsule endoscopic study showed that ASA-induced small intestine injury is milder than that of other NSAIDs.^22^ As we mentioned above, animal models of ASA-induced small intestine injury have been challenging to establish. Nonoyama et al. succeeded in artificially triggering small intestine injury by exposing the rat duodenum directly to ASA.^23^ Although Lai et al. were able to simulate small intestine injury by administrating ASA continuously for 5 d,^24^ we could not reproduce their data, possibly because of difficult-to-evaluate factors such as the breeding environment.

NSAID-induced small intestine injury is correlated with increased intestinal permeability.^25^ Consistent with reports that a high-fructose diet increases intestinal permeability,^7^ we demonstrate here that a high-fructose diet is key to stably inducing small intestine injury, independent of breeding conditions (data not shown). The increase in the number of MPO-positive cells we observed in our model suggests the involvement of neutrophils, consistent with prior research that suggests neutrophils mediate indomethacin-induced intestinal injury.^26^

In this study, we examined the effect of PPI on ASA-mediated intestinal damage. To examine the effects of PPIs in detail, we focused on the analysis of the microbiota in the jejunum. Prior studies have shown that PPI causes dysbiosis, increasing the levels of *Lactobacillus and Streptococcus* in ileum and fecal samples^27-29^ while reducing *Bifidobacteria* levels in fecal samples;^30^ we made a similar observation (Figure S1, Figure 2, Figure S5). In the clinical study as shown in Figure 2a and S1, the relative abundance of *Akkermansia* was not changed. This is because samples were obtained from human feces, and subjects were allowed to eat anything except for fermented foods, which was different from our other studies in mice. Since PPIs reduce gastric acidity, they weaken the barrier against upper respiratory tract and oral commensals such as *Streptococcus. Lactobacillus* is reported to increase after PPI administration, although the mechanism by which this occurs is unclear.^27,29^ It may be caused by the interactions of other gut microbiota that are affected by PPI administration. Interestingly, the combined use of PPI and ASA resulted in an increase in serum endotoxin levels (Figure 2e). It has been reported that endotoxin induces procoagulant activity and increases the risk of thrombosis and atherosclerosis.^31^ Furthermore, indomethacin promoted endotoxin-induced procoagulant activity.^32^ The other factor that promotes thrombosis is trimethylamine (TMA), a metabolite produced by gut microbiota. TMA is oxidized to trimethylamine N-oxide (TMAO) after TMA is absorbed from the intestine. High levels of TMAO increase the risk of thrombosis through platelet hyperactivity, which in turn promotes atherosclerosis.^33^ Our study demonstrated that the endotoxin levels of the PPI alone group were not increased (Figure 2e) despite PPIs changing the composition of gut microbiota (Figure S1, S2). This finding suggests that the severe enteropathy caused by ASA and PPI resulted in barrier dysfunction, which might lead to increase levels of endotoxin and TMAO in the bloodstream.

It has been reported that an expansion of *A. muciniphila* in the colon following fiber deprivation has been reported.^34^ It causes gut barrier dysfunction as a result of the mucolytic effect of mucus-degrading bacteria, which is a similar idea in our study. It is important to note that this is the first study to report the abnormal growth of *A. muciniphila* in the murine jejunum. Since *A. muciniphila* is an obligate anaerobe, it is unlikely to inhabit the upper small intestine; in fact, it was not detected in the jejunum in the vehicle-only control group. However, the relative abundance of *Akkermansia* in the jejunum was much higher in the mice that received PPI. Because its growth is optimal at pH 6.5,^17^ *A. muciniphila* may be able to inhabit the jejunum following the use of a PPI. In contrast, *Akkermansia* levels were not increased in the basal diet fed group despite omeprazole administration (Figure S3C). This finding indicates that, together with the administration of PPIs, diet is a key determinant of *Akkermansia* overgrowth. A previous study reported that levels of *A. muciniphila* decreased due to omeprazole treatment;^35^ however, the study involved the investigation of murine fecal samples, which differentiates it from our study. How *Bifidobacteria* influences the level of *Akkermansia* in the gut remains unclear. In our study, the administration of *Bifidobacterium bifidum* G9-1 decreased *Akkermansia* in the jejunum (Figure 3a); this has also been seen in the colon.^36^ However, it is unclear whether there is a relationship between *Bifidobacteria* and *Akkermansia* and their respective colonization abilities. The administration of *Bifidobacterium bifidum* G9-1 might indirectly decrease *Akkermansia* through changing the composition of gut microbiota. Detailed mechanisms underlying this growth of *Akkermansia* in the jejunum remain to be elucidated in future investigations.

The intestinal mucus layer covers the intestinal surface and protects it from external threats. Thinning of the mucus layer is one reason for the increases in intestinal permeability. To our knowledge, this study is the first to observe aggravation of small intestinal permeability and thinning of the mucus layer by *Akkermansia* (Figure 3). Furthermore, since compromised mucus protection can allow the transport of endotoxins and bacteria into blood vessels,^25^ we consider *Akkermansia* to be involved in ASA-induced injury caused by PPI. In prior studies, the administration of *Akkermansia* to obese mice ameliorated obesity by improving the colon barrier.^37–39^ These effects were not observed in the small intestine in our study, possibly because the mucus layer of the small intestine is much thinner than that of the colon.^40^ *A. muciniphila* produces short-chain fatty acids (SCFAs) including butyrate as a result of mucus degradation.^41^ As will be mentioned later, SCFAs play an important role in the gut barrier. However, we speculate that the overgrowth of *A. muciniphila* is limited to the jejunum, and not the colon in this study. Therefore, the amount of SCFAs production in our model may be small, which would preclude observation of any positive effects in our model.

Here, we speculate that G9-1 repairs the damage in two ways. First, the intestinal mucosa repair process includes restitution and regeneration. Restitution requires TFF3,^14^ a peptide secreted from goblet cells that is primarily found in the intestinal tract. Goblet cells protect and repair the intestinal mucosa and promote the maintenance of the mucus layer through an interaction with MUC2.^42^ In this study, the increasing trend of the number of goblet cells and expression levels of *Tff3* were observed in mice fed a basal diet and treated with G9-1 (Figure S6). Thus, it is possible that G9-1 not only increases the expression of *Tff3*, but also stimulates mucin secretion to thicken and stabilize the mucus layer. In this study, G9-1 was not associated with the regeneration because cell proliferation markers such as BrdU and Ki67 and *Cox-2* expression in the ASA+PPI+G9-1 and ASA+Akk+G9-1 groups were not increased (Figure 4a, b, c). This result may at first seem paradoxical, since ASA inhibits COX-1 and COX-2. In fact, our data demonstrated that ASA decreased PGE2 levels (Figure 4d). However, it may be explained by the single administration of ASA and the short time from the administration to sacrifice; this essentially recapitulates the acute inflammatory response, where cell proliferation was promoted to repair mucosae. Therefore, ASA did not play an important role in the inhibition of COX-2 with regard to the cell proliferation in this study. Additionally, there are some reports demonstrating that NSAIDs upregulate *Cox-2* expression in NSAID-induced enteropathy models.^43,44^

Second, G9-1 regulates the immune system by inducing the differentiation of small intestinal LPL cells to Treg cells, triggering an anti-inflammatory response through IL-10 (Figure 5). Differentiation of Tregs is induced by probiotics when metabolites that comprise the cell wall, or short-chain fatty acids (SCFAs), bind to the host’s receptors,^45–47^ which might improve intestinal permeability. *Bifidobacteria* can also produce acetate, which is associated with an intestinal protective effect.^48^ In this study, the administration of pasteurized G9-1 significantly suppressed the increase in intestinal permeability. The reduction was not significant when the acetate solution was administered (*p* = .05), although the average was almost equivalent to the ASA+PPI+pasteurized G9-1 group (Figure S7). This result suggests that G9-1 plays an important role in Treg induction, either by producing acetate, or through the presence of G9-1 structural proteins such as the cell wall.

The involvement of TGF-β and butyrate in the differentiation of Tregs is widely known. TGF-β promotes the expression of Foxp3, and induces the differentiation to Tregs. However, in the presence of IL-6, differentiation of naïve T cells to Th17 cells is promoted and Treg differentiation is suppressed.^49,50^ In our study, the administration of G9-1 reduced *Il-6* levels and increased *Tgf-β* levels (Figure 5). These results suggest that G9-1 promotes the differentiation of naïve T-cells to Tregs by suppressing their differentiation to Th17 cells.

On the other hand, butyrate suppresses the activation of histone deacetylase to induce the differentiation of naïve T-cells to Tregs and triggers an anti-inflammatory response. Although G9-1 does not produce butyrate directly, the concentration of butyrate in the cecum was increased by the administration of G9-1 compared to the vehicle group (Figure S8). A prior study has shown that G9-1 improves gut dysbiosis and increases the levels of butyrate-producing bacteria in the intestine.^51^ Butyrate levels were also increased by G9-1 in our study; we suspect that this is due to changes in the gut microbiota composition. There were no significant differences between the ASA+PPI and the ASA+PPI+G9-1 groups. This may be because *A. muciniphila* was increased in the ASA+PPI group, where it also produced acetate by degrading the mucus layer in the jejunum.^17^ It is reported that butyrate-producing bacteria use acetate as a co-substrate to produce butyrate.^52^ However, the mucus layer in the jejunum may be too thin to produce enough SCFAs by *A. muciniphila*.

**Figure 6.**
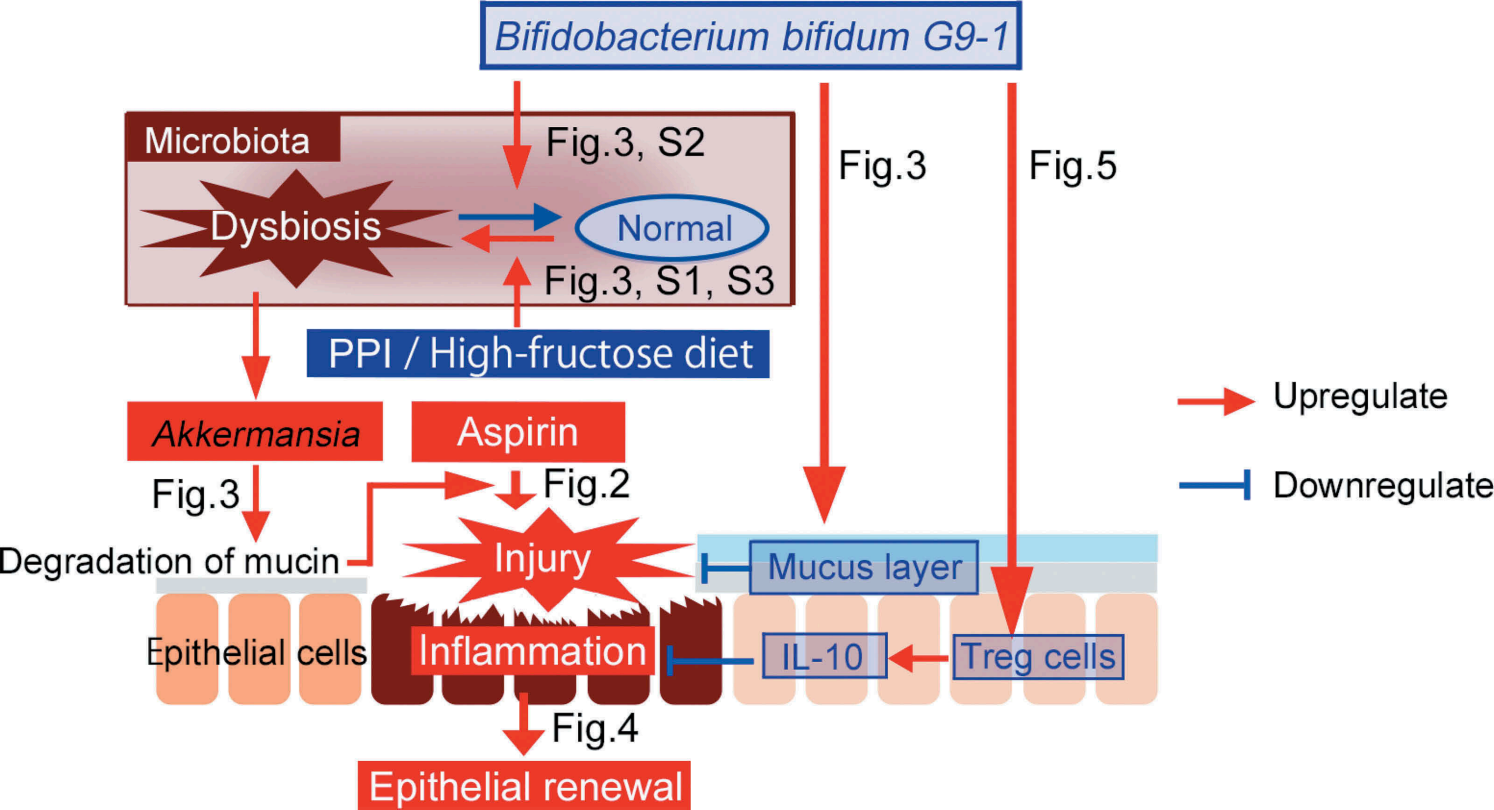
Diagram of the proposed mechanism. Both the administration of PPIs and a high-fructose diet lead to increases in *Akkermansia* in the jejunum, which is associated with mucin degradation. As a result, this exacerbates ASA-induced small intestine injury. Epithelial renewal is promoted when intestinal mucosa is injured. G9-1 suppresses the growth of *Akkermansia* in the jejunum, which suppresses mucin degradation, along with an increase in the expression of genes that are protective of epithelial cells. Furthermore, G9-1 induces Treg differentiation, which leads to suppression of inflammation in the small intestine.

In conclusion, ASA-induced small intestine injury was clearly observed in mice fed a high-fructose diet. PPI exacerbated this by causing a thinning of the mucus layer, which was directly caused by gut dysbiosis, characterized mainly by the overgrowth of *A. muciniphila*. In contrast, treatment with G9-1 suppressed the exacerbation of small intestine injury caused by PPI. G9-1 is therefore a promising therapeutic approach for gut dysbiosis in the jejunum, where it stabilizes the mucus layer by increasing goblet cells, and promotes differentiation to Tregs (Figure 6).

There are several limitations to this study. The primary difference is that mouse and human microbiota and immune systems have several differences, and therefore we cannot claim to have fully elucidated the situation in humans. Since jejunum samples were not obtained from human subjects, we could not confirm the overgrowth of *A. muciniphila* in humans. Moreover, we have insufficient evidence to conclude whether the effects of ameliorating small intestine injury are specific to G9-1. Additional studies are therefore required to ascertain whether other probiotics have similar effects.

## Materials and methods

### Ethics approval

The clinical study was conducted based on the Declaration of Helsinki and accepted by the Yokohama City Hospital ethical committee (Project Number: A151126008). Animal studies were conducted by following the guidelines of the Ethics Committee of the Medical school of Yokohama City University as well as the animal experiment guidelines of Biofermin Pharmaceutical, and accepted by the Animal Care and Use Committee of Yokohama City University and Experimental Animal Care and Use Committee of Biofermin Pharmaceutical Co., Ltd. (Acceptance Number 131–002).

### Bacteria

*Bifidobacterium bifidum* G9-1, *Akkermansia muciniphila* ATCC BAA835 ^T^, and *Lactobacillus reuteri* DSM 20016 ^T^ were obtained from the Culture Collection of Biofermin R&D Center (Kobe, Japan), ATCC (Manassas, VA, USA) and DSMZ (Braunschweig, Germany), respectively.

*Bifidobacterium bifidum* G9-1 and *Lactobacillus reuteri* DSM 20016 T were cultured for 18 h at 37°C in GAM broth (Nissui Pharmaceutical Co., Ltd., Tokyo, Japan) supplemented with 0.7% glucose and 0.1% Tween-80. *Akkermansia muciniphila* ATCC BAA835 was cultured for 18 h at 37°C in BHI broth (Kanto Kagaku Co., Ltd., Tokyo, Japan) supplemented with 0.5% L-cysteine hydrochloride (FUJIFILM Wako Pure Chemical Co., Ltd., Osaka, Japan). Before administration, bacteria were washed twice with phosphate-buffered saline (PBS), and the pellets obtained after low-speed centrifugation were stored at −80°C until use.

### Clinical study

Prior to the animal study, we conducted a clinical study in humans to address whether PPI causes gut dysbiosis. Nineteen healthy male subjects (20–33 years old) were administered omeprazole (20 mg/day) (AstraZeneca) orally for 14 days. Subjects collected fecal samples at home before and after drug administration. During the study, subjects were instructed to limit their consumption of fermented products.

### Mouse model

Male C57BL/6JJcl mice were purchased from CLEA Japan (Tokyo, Japan). All mice were housed in the specific pathogen-free environment (22–24°C, 60% humidity, 12 h light and 12 h dark cycle). Between 2 and 4 mice were co-housed in each cage. Before the experiments, mice were adapted to the environment for a week, and then randomly allocated to the experimental groups.

#### Aspirin-induced small intestine injury model

Basal (MF, Oriental Yeast Co., Ltd.) or a 60% fructose diet (D00111301Y, Research diets) was fed to six-week-old mice for 9 weeks. On the final day, mice were fasted 6 h before sacrifice. ASA (200 mg/kg) or 5% gum arabic as a vehicle (both FUJIFILM Wako Pure Chemical Corporation) was administered orally 3 h before sacrifice and 600 mg/kg of FITC-dextran (average molecular weight 4,000) (Sigma-Aldrich) was administered orally 1 h before sacrifice. Between 10 and 11 mice were used to assess the injury-forming rate as shown in Figure 1c.

#### The effect of omeprazole and G9-1 on the injury

A 60% fructose diet was fed to six-week-old mice for 9 weeks. Mice were divided into five treatment groups as shown in Figure 2b. During the trial period, 20 mg/kg omeprazole or 0.9% NaCl as vehicle was administered intraperitoneally once daily. G9-1 (1 × 10^9^ colony forming units (CFU)/day) or PBS as vehicle was administered orally for 1 week before sacrifice. On the last day, mice were fasted 6 h before sacrifice. ASA (200 mg/kg) or 5% gum arabic (FUJIFILM Wako Pure Chemical Co., Ltd.) as the vehicle was administered orally 3 h before sacrifice, after which 750 mg/kg of FITC-dextran (average molecular weight 4,000) was administered orally 1 h before sacrifice. Between 7 and 11 mice were used for evaluating the histological score as shown in Figure 2d, 5–8 mice were used for measuring serum endotoxin as shown in Figure 2e, 5–8 mice were used for measuring intestinal permeability as shown in figure 2f, and 6–8 mice were used for the analysis of *Akkermansia* and *Bifidobacteria* as shown in Figure 3a. Between 5 and 10 mice were used for investigating the expression levels of inflammatory cytokines and chemokines as shown in Figure 5a, 5–7 mice were used for investigating expression levels of *Il-10, Tgf-*β, and *Foxp3* as shown in Figure 5b and Figure 5d, and 4–5 or 8–10 mice were used for flow cytometry analysis, as shown in Figure 5c and d.

#### *The effect of* A. muciniphila *and G9-1 on the mucus layer and goblet cells*

A 60% fructose diet was fed to six-week-old mice for 9 weeks, and divided into six treatment groups as described in Figure 3d. Either 20 mg/kg of omeprazole, *A. muciniphila* (1 × 10^9^ CFU), or 0.9% NaCl as the vehicle was administered once daily during the treatment period. G9-1 or PBS as vehicle was administered orally for 1 week before sacrifice. On the last day, mice were fasted 6 h before sacrifice. ASA or 5% gum arabic as vehicle was administered orally 3 h before sacrifice. Five or six mice were used for assessing the thickness of mucus layer and the number of goblet cells as shown in Figure 3e and g. Between 8 and 11 mice were used for investigating the expression levels of *Tff3* in the jejunum as shown in Figure 3h. Five mice were used for the BrdU incorporation and Ki67 staining study as shown in Figure 4a and b. Six or seven mice were used for investigating the expression levels of *Cox-2* as shown in Figure 4c. Seven mice were used for PGE2 ELISA as shown in Figure 4d.

### Gnotobiotic trial

We conducted this trial to confirm that *A. muciniphila* is a key factor in the exacerbation of small intestine injury. Germ-free male C57BL/6NJc1 mice were purchased from CLEA Japan (n = 8 for each group) and housed in a germ-free environment. Six-week-old mice were fed a 60% fructose diet and divided into four treatment groups as shown in Figure 3c. *A. muciniphila* and *Lactobacillus reuteri* were suspended in PBS at 2 × 10^8^ CFU/0.2 mL. On the first day, the mice received either *A. muciniphila* or *Lactobacillus reuteri* orally. From the second day, 20 mg/kg omeprazole was administered intraperitoneally every day. G9-1 at 1 × 10^9^ CFU/day was administered orally to the Akk+G9-1 group from the second day until the end of the study. One hour before sacrifice, 750 mg/kg FITC-dextran (average molecular weight 4,000) was administered orally.

### Pathology

For hematoxylin-eosin (HE) staining, samples were fixed with 10% formalin overnight. Histopathology of the jejunum was assessed using the scoring system of Chiu et al.^53^ (Table 1) in a blinded manner by two independent investigators to prevent bias; histology was also subject to a final check by a pathologist. Six or more points of injured areas for each sample were translated into scores, and the average of the scores was used for the assessment.

**Table 1.** Histopathological score.

Grade	
0	Normal mucosal villi.
1	Development of subepithelial Gruenhagen’s space, usually at the apex of the villus.
2	Extension of the subepithelial space with moderate lifting of epithelial layer from the lamina propria.
3	Massive epithelial lifting down the sides of villi. A few tips may be denuded.
4	Denuded villi with lamina propria and dilated capillaries exposed.
5	Digestion and disintegration of lamina propria; hemorrhage and ulceration.

For PAS staining, jejunum samples were fixed with Carnoy solution in order to measure the thickness of the mucus layer, then transferred to 70% ethanol. The thickness of the mucus layer was measured using approximately 124 points per sample, and it was assessed using NIH ImageJ software.^54^ The number of goblet cells in six visual fields per sample was counted, and the score was the number of goblet cells divided by the number of villi. The thickness of the mucus layer and the counts of goblet cells were assessed only in undamaged areas of the jejunum to allow uniform comparisons between groups.

### Immunofluorescent staining for myeloperoxidase (MPO)

To observe inflammatory cell infiltration in the small intestine, we stained for MPO-containing cells using paraffin-embedded tissue samples. An anti-MPO antibody (ab9535, Abcam) was used as the primary antibody, followed by a goat anti-rabbit IgG (H+L) cross-adsorbed secondary antibody labeled with Alexa Fluor 488 (Thermo Fisher Scientific).

### Assays for BrdU and Ki67

To investigate epithelial cell proliferation, we performed immunohistochemical staining for BrdU and Ki67. Prior to harvesting samples, we intraperitoneally administered 3 mg of BrdU (550891, BD Biosciences) 1 h before sacrifice.

For the staining of BrdU and Ki67, jejunum samples were fixed with 10% formalin overnight. An anti-mouse BrdU (B35128, Invitrogen), anti-mouse Ki-67 (ab264429, Abcam) were used as the primary antibodies. Histofine Mouse Stain Kit (Nichirei) for BrdU, Histofine Simple Stain Mouse MAX-PO(R) (Nichirei) for Ki67 were used as the secondary antibodies. The total number of positive cells from 50 crypts was used to assess the proliferative rate of cells.

### Blood FITC-dextran levels

After mice were anaesthetized, blood samples were immediately taken from inferior vena cava. They were centrifuged for 15 min at 800 × *g* at 4°C. The plasma was used to quantify intestinal permeability. Blood FITC-dextran levels were assessed by fluorescence measured with a plate reader (Promega, Madison, WI, USA or TECAN, Männedorf, Switzerland) with excitation/emission at 485/535 nm.

### Endotoxin measurements

Serum samples were diluted two-fold and then placed on a heat block for 5 min. The endotoxin levels in the samples were then quantified using a *Limulus* Amoebocyte Lysate Chromogenic Endpoint Assay (Hycult Biotech, Noord-Brabant, Netherlands).

## PGE2 ELISA

To quantify PGE2 levels in the jejunum samples, an ELISA was performed. Samples were obtained via extraction. First, 20 mg of jejunum sample was homogenized with 0.1 M phosphate buffer at pH 7.4 containing 1 mM EDTA and 10 µM indomethacin, after which 2 M HCl was added. Homogenates were stored for 15 min at 4°C and then centrifuged for 10 min at 3,000 × *g*.

A C18 reverse-phase column (Sep-Pak Vac 3cc (200 mg) tC18 Cartridges, WAT054925, Waters, Milford, MA, USA) was washed with 3 ml of ethanol followed by 3 ml of deionized water. The samples were applied to the columns and washed with 3 ml of water, followed by 3 ml of 15% ethanol and 3 ml of hexane. Samples were eluted with 3 ml of ethyl acetate and then evaporated with a Speed Vac DNA 110 concentrator (Savant, NY, USA).

PGE2 quantification was performed using a Prostaglandin E2 ELISA kit (ab133021, Abcam) according to the protocols of the manufacturer.

### Quantitative RT-PCR

RNA extraction was performed with the miRNeasy Mini Kit or RNeasy Midi kit (Qiagen). Reverse transcription to cDNA was conducted using the High Capacity RNA-to-cDNA Kit or SuperScript® VILO™ MasterMix (both Thermo Fisher Scientific) according to the manufacturer’s instructions. The mRNA expression levels were measured using the Taqman method (FastStart Universal Probe Master (ROX) (Roche Applied Sciences) or TaqMan® Fast Advanced Master Mix (Thermo Fisher Scientific) and a StepOnePlus Real-Time PCR System or a QuantStudio® 3 RealTime PCR system (both Thermo Fisher Scientific). The primer sequences are shown in Supplementary Information Table 1. *Il-10, Tgf-β1, Foxp3*, and *Cox-2* (*Ptgs2*) were purchased from Sigma-Aldrich. *Tff3*, glyceraldehyde-3-phosphate dehydrogenase (*Gapdh*), and beta-actin (*Actb*) were purchased from Thermo Fisher Scientific. *Actb* was used as a housekeeping gene for *Il-10, Tgf-β1, Foxp3*, and *Cox-2. Gapdh* was used as a housekeeping gene for *Tff3*.

### Transfection of siRNA

We purchased siRNA against *Tgf-β1* (Silencer Select siRNA, s75041) and scramble (Silencer Select Negative Control No. 1 siRNA) from Thermo Fisher Scientific. The 250 nmol of siRNA was diluted into 338 µl of PBS. The siRNA was encapsulated with the Hemagglutinating Virus of Japan Envelope (HVJ-E) (GenomeOne-si, Ishihara Sangyo) according to the manufacturer’s protocol. siRNA (7.4 nmol) was transfected by intraperitoneal injection 48 h and 12 h before sacrifice.

### Quantification of cytokines & chemokines

Serum cytokine and chemokine levels were quantified using the Bio-Plex mouse cytokine GI 23 panel (Bio-Rad, Hercules, CA, USA).

### Lamina propria lymphocyte (LPL) isolation

LPLs were isolated according to previously published protocols^55,56^ with modifications. The upper 2/5 of the small intestine was removed and then opened. The tissue was washed with 30 mM EDTA on ice and the intestinal epithelium was discarded. RPMI 1640 (Nacalai Tesque) was added to the remaining tissue along with 50 mg/mL collagenase D and 1 mg/mL DNAase (Roche), then incubated at 37°C for 16 min. The supernatant containing released cells was passed through a 70-µm strainer and suspended in 40% Percoll, after which the cell suspension was layered onto 80% Percoll and centrifuged for 12 min at 700 × *g*. Cells were collected and washed with 10% RPMI, then centrifuged for 4 min at 800 × *g* at 4°C.

### Flow cytometry

Isolated LPLs were used to examine CD25^+^ Foxp3^+^ cells in the upper small intestine. Nonspecific staining was blocked by TruStain FcX (anti-mouse CD16/32, BioLegend). Cells were then stained with the following fluorescently labeled antibodies: anti-CD3 (17A2, BioLegend), anti-CD4 (GK1.5, Thermo Fisher Scientific), anti-CD25 (PC61.5, Thermo Fisher Scientific), and anti-FOXP-3 (FJK-16s, Thermo Fisher Scientific). Foxp3/Transcription Factor Fixation/Permeabilization Concentrate and Diluent (Thermo Fisher Scientific) was used to detect intracellular Foxp-3. Staining was analyzed using Attune NxT (Thermo Fisher Scientific, Waltham, MA, USA).

### Analysis of microbiota

Jejunum fecal samples were collected by washing the intra lumen of the jejunum with 8 ml of sterile 0.9% NaCl. DNA extraction was performed as previously described^57^ and DNA was stored at −80°C until use. Analysis of the V3-V4 region of bacterial 16 S rDNA was performed as previously described with minor modifications.^58^ Briefly, amplicons of the V3-V4 region of 16S rDNA containing unique indices incorporated by the Illumina Nextera XT Index kit (Illumina) were purified by AMPure XP beads. Each purified barcoded library was diluted to 4 nM using 10 mM Tris-HCl (pH 8.0), after which equal concentrations of each library were mixed to provide a final concentration of each of 10 pM prior to multiplex sequencing. Following this, the libraries were mixed with 40% PhiX control DNA, the final concentration of 10 pM. Sequencing was performed using a 2 × 250-bp paired-end run on a MiSeq platform with MiSeq Reagent Kit v2 chemistry (Illumina).

For the mouse model, sequence analysis was conducted using the Quantitative Insights Into Microbial Ecology (QIIME)1 pipeline (http://qiime.org/),^59^ as previously described.^60^ First, raw read data have been reduced to 30000 to reduce the burden on the computer and shorten the analysis time. Randomly obtained raw reads (30000) of each sample were merged by fastq-join. Subsequently, low-quality reads were filtered and a chimera-check was conducted. After read-merging and eliminating low-quality reads, 5,000 high-quality reads per sample were randomly chosen to minimize the overestimation of species richness in the clustering due to intrinsic sequencing error,^61^ and operational taxonomic units (OTUs) for the total high-quality reads were constructed with a 97% identity threshold. Representative reads from each OTU were then assigned to the 16S rRNA gene database with ≥ 97% identity (NCBI taxonomy database). Beta diversity was estimated by computing the Bray–Curtis dissimilarity and weighted UniFrac distance between samples, using a phylogenetic tree-based metric.^62^ In the clinical study, sequences were merged using CLC Genomics Workbench (CLC Bio, Aarhus, Denmark), analyzed by homology using Local RDPClassifier (World Fusion, Tokyo, Japan) and assigned to genera using Metagenome@KIN (World Fusion).

### Cecum butyrate measurements

Cecum butyrate concentrations were measured by Technocrat Lab Ltd. (Shizuoka, Japan).

### L. reuteri *mucin utilization ability*

*L. reuteri* was cultured on a medium to observe changes in pH as an indicator of bacterial growth because bacteria produce short-chain fatty acids, such as lactate, which results in decreased pH. Tryptone Soya Broth without dextrose (TSB, Sigma-Aldrich) with 0.2% mucin from porcine Type Ⅱ stomach (Sigma-Aldrich) was spread on 1% glucose containing TSB agar and incubated under aerobic or anaerobic conditions to verify sterility. The medium without mucin was used as a negative control. Next, *L. reuteri* that had been cultured with 1% glucose containing TSB medium at 37°C for 24 h was added to each medium and incubated at 37°C for 18 h. The pH of the medium was measured with a pH meter (D-72, HORIBA, Ltd., Kyoto, Japan).

### Statistical analyses

All statistical analyses were performed with EZR (Saitama Medical Center, Jichi Medical University, Saitama, Japan),^63^ and statcel4 (OMS publishing Inc., Saitama, Japan).

Data are shown as means ± standard error. To compare data from two different groups, Student’s *t*-test or Welch’s *t*-test were used for the normally distributed data, the Wilcoxon rank-sum test was used for the non-normally distributed data. Wilcoxon signed-sum test was used to compare two related samples that are not normally distributed as shown in Figure 2a. Fisher’s exact tests were performed to assess the incidence of small intestine injury as shown in Figure 1c. To compare data obtained from three or more groups, the Tukey–Kramer test, Dunnett’s test, or Steel-Dwass test were employed according to the distribution of the data. PERMANOVA test was performed when the clustering shift was assessed. A *p* value of less than 0.05 was considered significant.

## Supplementary Material

Supplemental Material
