# Supplementary material for: The protective effect of Bifidobacterium bifidum G9-1 against mucus degradation by Akkermansia muciniphila following small intestine injury caused by a proton pump inhibitor and aspirin
Source: Gut Microbes. 2020 Jun 9;11(5):1385–404. doi: 10.1080/19490976.2020.1758290 (PMC7527075; doi:10.1080/19490976.2020.1758290)
Supplement: Supplemental Material [file KGMI_A_1758290_SM3239.zip › Gut_microbes_Supplementary Information Table 1__revise.docx]

**Supplementary Information Table 1** Primers used in gene expression assays.

| **Assay Target** |  | |
| --- | --- | --- |
| *Il-10* | **Probe** | TCTTCACCTGCTCCACTGCCTTGCTCT |
|  | **Forward** | TGAGGCGCTGTCATCGATTTC |
|  | **Reverse** | CCTTGGTCTTGGAGCTTATTAAAATC |
| *Tgf-β* | **Probe** | TTCAGCCACTGCCGTACAACTCCAGT |
|  | **Forward** | CCCACTGATACGCCTGAGTG |
|  | **Reverse** | AAGCCCTGTATTCCGTCTCC |
| *Foxp3* | **Probe** | CTGTGCTCCAAGTGCGTCCACTGGA |
|  | **Forward** | GCATCAGCTCTCCACTGTGG |
|  | **Reverse** | AGCAGAAGGTGGTGGGAGG |
| *Cox-2* （*Ptgs2*） | **Probe** | TGATCGAAGACTACGTGCAACACCTGAGC |
|  | **Forward** | CAGACTCATACTCATAGGAGAGACTA |
|  | **Reverse** | GGAACTGCTGGTTGAAAAGGAG |
| *Tff3* | **Catalog number** | Mm00495590_m1 |
| *Gapdh* |  | Mm99999915_g1 |
| *Actb* |  | 4352341E |
