# Supplementary material for: The protective effect of Bifidobacterium bifidum G9-1 against mucus degradation by Akkermansia muciniphila following small intestine injury caused by a proton pump inhibitor and aspirin
Source: Gut Microbes. 2020 Jun 9;11(5):1385–404. doi: 10.1080/19490976.2020.1758290 (PMC7527075; doi:10.1080/19490976.2020.1758290)
Supplement: Supplemental Material [file KGMI_A_1758290_SM3239.zip › Supplementary Captions.docx]

**Figure S1**

Change in the gut microbiota profile in humans. Omeprazole (20 mg/kg) was administered to 19 healthy male human subjects for 14 days. (A) Relative abundance of the dominant genera. (B) Bray-Curtis dissimilarity before and after omeprazole treatment.

**Figure S2**

Weighted UniFrac distance analysis. The gut microbiota in the jejunum of mice in the different treatment groups was analyzed.

**Figure S3**

Relative abundance of *Akkermansia* and *Bifidobacteria* in the jejunum of mice. Male C57BL/6JJcl mice (2 or 3) were housed in each cage. (A) Levels of *Akkermansia* prior to being fed a high-fructose diet and drugs (six-week-old mice) compared to the vehicle control group (n = 8 for each group). “Before” in this figure indicates “before being fed a high-fructose diet and drugs.” (B) Levels of *Akkermansia* and *Bifidobacteria* in the vehicle control and ASA alone groups (n = 8 for each group). (C) Levels of *Akkermansia* in the mice fed a basal diet which was intraperitoneally administered with either 0.9% NaCl as vehicle or 20mg/kg of omeprazole (n = 7–8 per group). Data are means ± SEM. *p < 0.05, **p < 0.01, N.S., not significant, Wilcoxon rank-sum test (A–C).

**Figure S4**

*L. reuteri* mucin utilization ability. *L. reuteri* was cultured on a medium with 0.2% mucin. The medium without mucin was used as a negative control. Mucin utilization was assessed by measuring the reduction in pH.

**Figure S5**

Relative abundance of *Bifidobacteria* in the caecum. Four male C57BL/6JJcl mice were housed in each cage. Six-week-old mice were fed a 60% fructose diet and administered vehicle or PPI for 9 weeks (n = 8 for each)*.* Data are means ± SEM. Wilcoxon rank-sum test was used.

**Figure S6**

Goblet cells and *Tff3* gene expression levels in the jejunum of mice fed a basal diet (n = 4–5 per group). Male C57BL/6JJcl mice (2 or 3) were housed in each cage. Six-week-old mice were fed a basal diet and G9-1 (1 × 10⁹ CFU/head) was daily administered orally one week before sacrifice until study end. Data are means ± SEM. Student’s *t*-test was used.

**Figure S7**

Effect of pasteurized *Bifidobacterium bifidum* G9-1 and acetate on small intestinal permeability. The method is shown in Figure 2B. Pasteurized G9-1 or 0.15 M acetic acid solution was administered for one week before the end of the study (n = 8–10 per group). Data are means ± SEM. *p < 0.05, Dunnett’s test.

**Figure S8**

Caecum concentrations of butyrate (n = 8–10 per group). The method is shown in Figure 2B. Data are means ± SEM. *p < 0.05, Steel-Dwass test.
