# Supplementary figures and images for: The protective effect of Bifidobacterium bifidum G9-1 against mucus degradation by Akkermansia muciniphila following small intestine injury caused by a proton pump inhibitor and aspirin
Source: Gut Microbes. 2020 Jun 9;11(5):1385–404. doi: 10.1080/19490976.2020.1758290 (PMC7527075; doi:10.1080/19490976.2020.1758290)

(A)

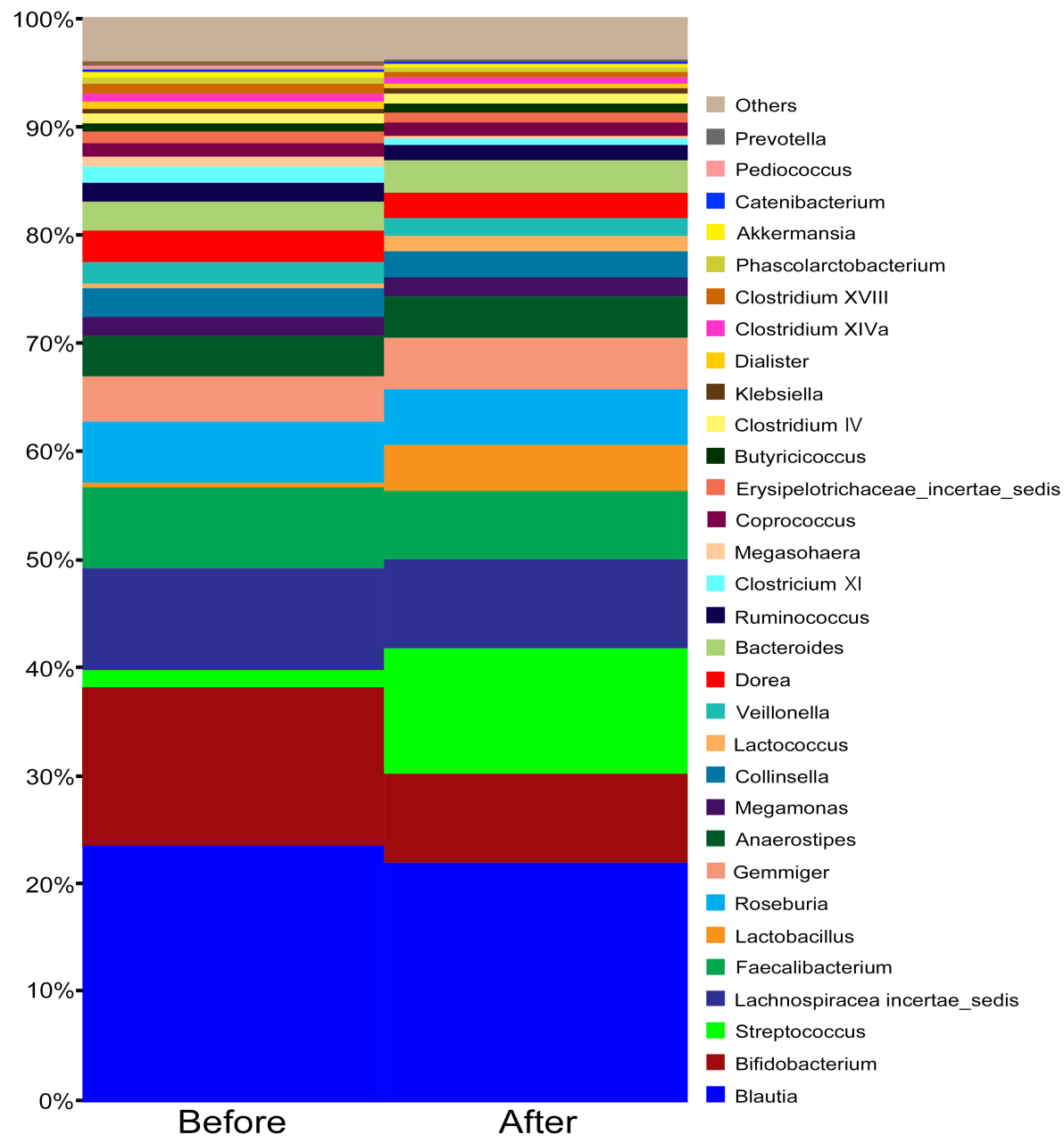

(B)

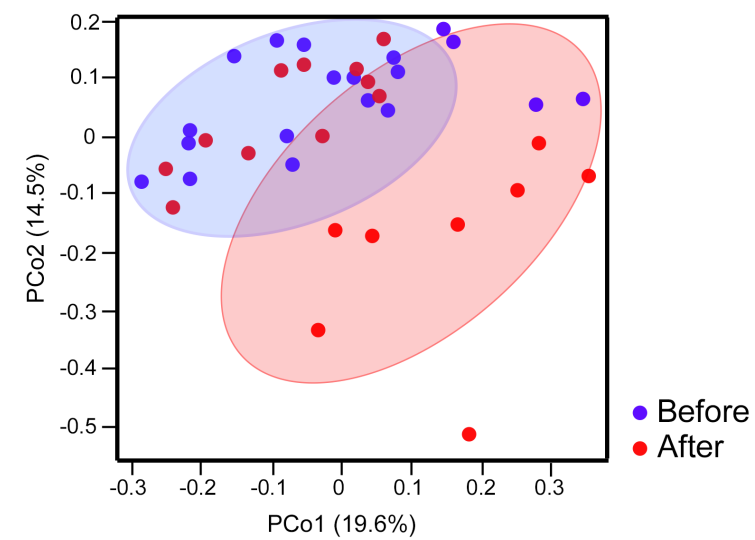

Supplement: Supplemental Material [file KGMI_A_1758290_SM3239.zip › Fig.S1.pdf]

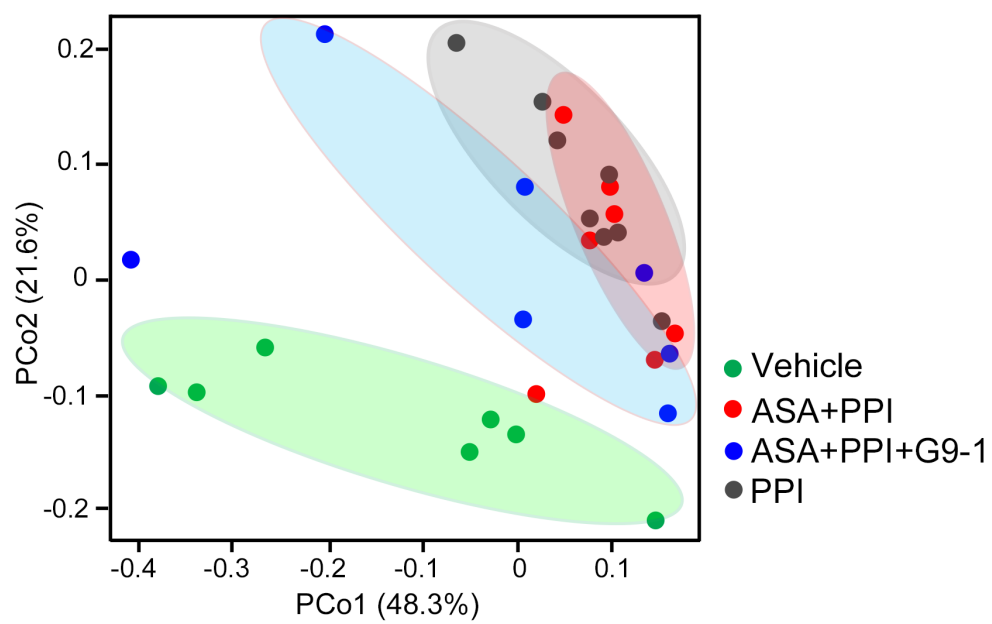

Supplement: Supplemental Material [file KGMI_A_1758290_SM3239.zip › Fig.S2.pdf]

(A)

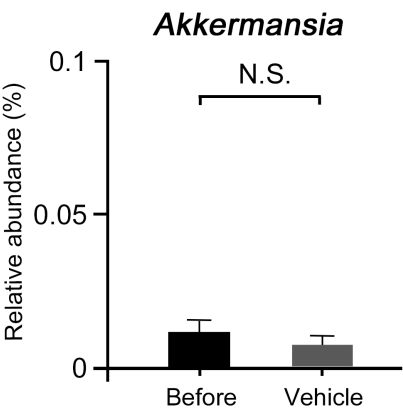

(B)

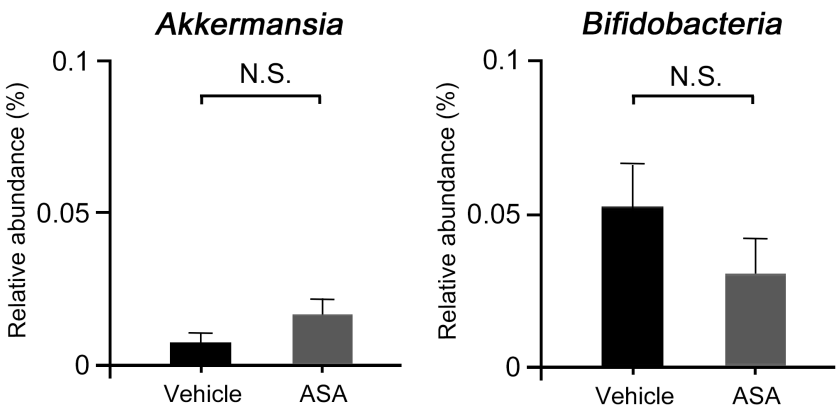

(C)

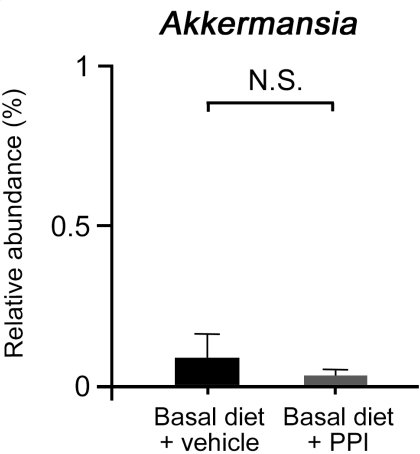

Supplement: Supplemental Material [file KGMI_A_1758290_SM3239.zip › Fig.S3.pdf]

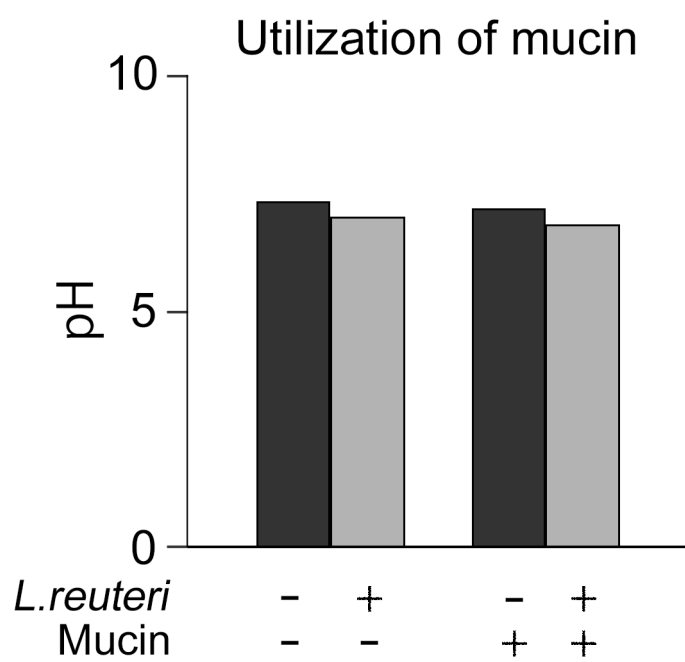

Supplement: Supplemental Material [file KGMI_A_1758290_SM3239.zip › Fig.S4.pdf]

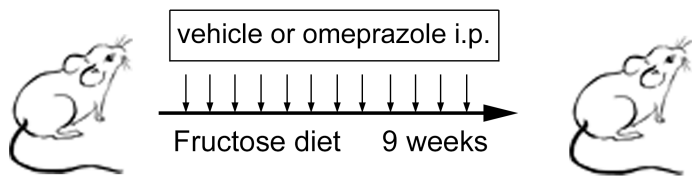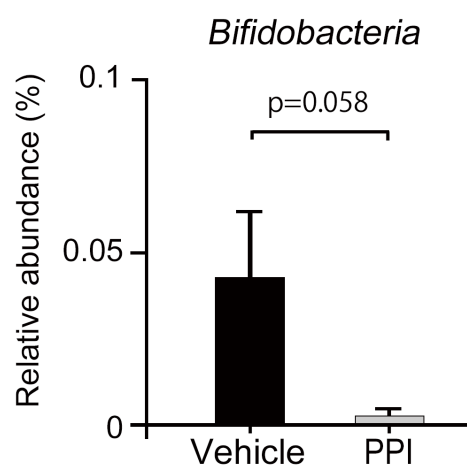

Supplement: Supplemental Material [file KGMI_A_1758290_SM3239.zip › Fig.S5.pdf]

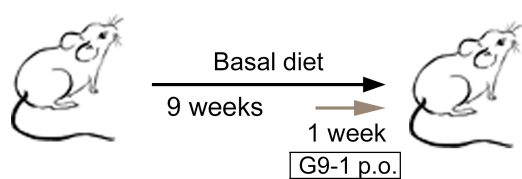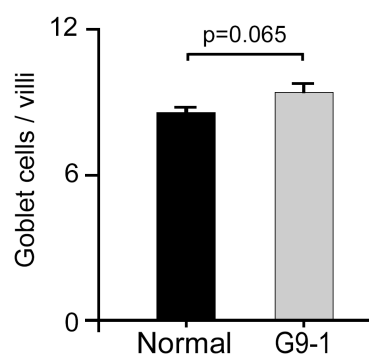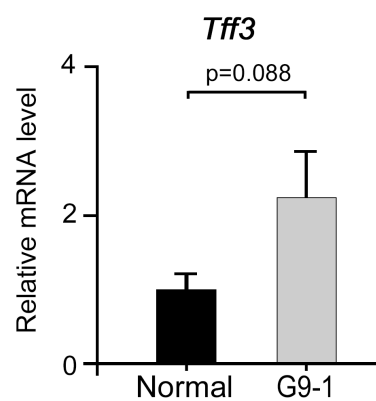

Supplement: Supplemental Material [file KGMI_A_1758290_SM3239.zip › Fig.S6.pdf]

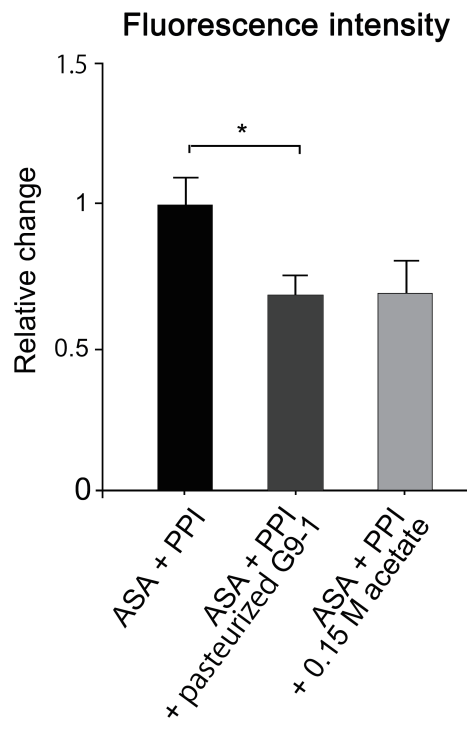

Supplement: Supplemental Material [file KGMI_A_1758290_SM3239.zip › Fig.S7.pdf]

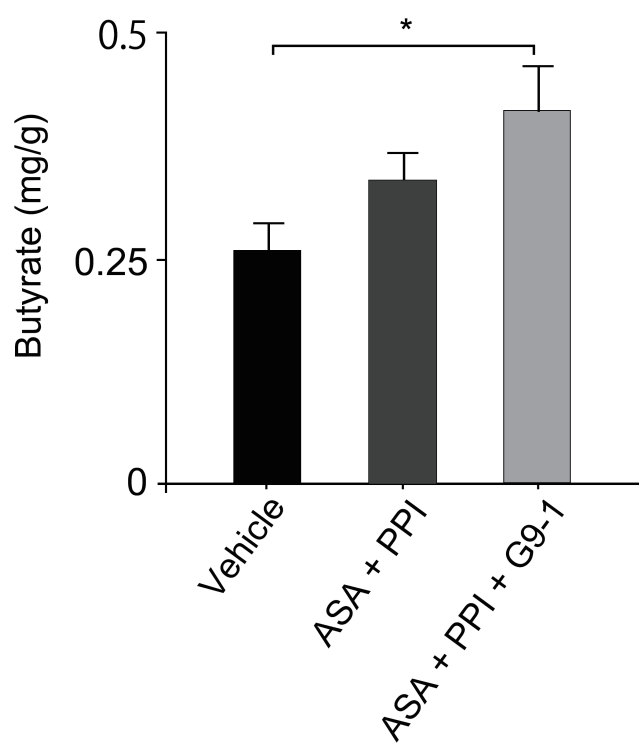

Supplement: Supplemental Material [file KGMI_A_1758290_SM3239.zip › Fig.S8.pdf]
